# Chemical Composition of Essential Oil, Phenolic Compounds Content, and Antioxidant Activity of *Cistus monspeliensis* from Northern Morocco

**DOI:** 10.1155/2021/6669877

**Published:** 2021-12-07

**Authors:** Sara Haida, Kaltoum Bakkouche, Abdelaziz Ramadane Kribii, Abderahim Kribii

**Affiliations:** Laboratory of Separation Processes, Environmental Chemistry Team, Department of Chemistry, Faculty of Science, Ibn Tofail University, Kenitra, Morocco

## Abstract

Currently, oxidative stress is one of the major problems that threatens human health. It is at the root of many diseases such as cancer. Despite the enormous efforts provided to combat this scourge, oxidative stress is still relevant and hence comes the need for research of new remedies especially from natural origin. For this purpose, the study of the antioxidant activity of extracts of *Cistus monspeliensis* from Morocco is a principal research objective. The phenolic extracts were obtained by maceration of the plant in a water/acetone mixture and then separated by liquid/liquid extraction with solvents of increasing polarity. The first phytochemical tests carried out on these extracts showed the existence of different families of phenolic compounds, such as flavonoids, tannins, and others. Assays for total polyphenols, flavonoids, hydrolysable, and condensed tannins were carried out by known colorimetric methods. The results of these assays have shown that the studied extracts are rich in phenolic compounds present in the plant in the form of flavonoids (69.81 ± 0.22 mg EQ/g DM), hydrolysable tannins (61.86 ± 0.89 mg ETA/g DM), and condensed tannins (70.05 ± 1.61 mg EC/g DM). The evaluation of the antioxidant activity is carried out by two different methods: the DPPH test (2,2-DiPhenyl-1-Picryl-Hydrazyl) and the FRAP test (Ferric Reducing Antioxidant Power). The results obtained show that the extracts of *Cistus monspeliensis* are active and have interesting antioxidant powers. In particular, the water/acetone (WAE) (IC_50_ = 0.079 mg/mL) and butanolic (BUE) (IC_0.5_ = 0.099 mg/mL) extracts are the most active with values comparable to that of ascorbic acid. The interesting results obtained in this study clearly show that *Cistus monspeliensis* originating from Morocco can be considered as a source of natural antioxidants. Therefore, the extracts of this plant deserve to be tested in the medicinal field, against cancer and cardiovascular diseases, and in food field as an additive and preservative.

## 1. Introduction

Natural products, especially those of plant origin, have always been a significant source of therapeutic agents due to their rich bioactive compounds content, known as polyphenols, since ancient times [1–3]. These compounds have received special attention for some years. They have a preventive role of cardiovascular and inflammatory diseases and also curative attributes of many diseases, such as cancer [2, 4–7]. These curative properties are due to their antioxidant and antimicrobial activity [8–10].

Our study comprises the survey of extracts from aromatic and medicinal plants originally from Morocco. We have chosen to study the plant *Cistus monspeliensis*, which appertains to the *Cistaceae* family and more precisely to the *Cistus* genus. This genus comprises about 20 species of shrubs; most of them are very aromatic and greatly appreciated by the perfume industry. *Cistus monspeliensis* occurs as shrubs generally growing around the Mediterranean and, in particular, in the north of Morocco (Rif region) on dry sunny soils, blooming on spring [11].

Previous pharmacological studies showed the interest in *Cistus monspeliensis* as an antimicrobial [12, 13], antioxidant [14], anti-inflammatory [15], and antidiabetic [16]. These previous results encouraged us to deepen the studies on this specie, studying extracts never before considered.

In addition to studying the chemical composition of *Cistus monspeliensis* essential oil, we were interested in determining the phenolic compounds present in these extracts as well as their antioxidant properties. The contents of total polyphenols, flavonoids, hydrolysable, and condensed tannins were determined by known colorimetric assay methods, and the antioxidant power of various extracts was achieved by the DPPH radical (2,2-DiPhenyl-1-Picryl-Hydrazyl) reduction method and by the iron reduction method FRAP (Ferric Reducing Antioxidant Power).

## 2. Materials and Methods

### 2.1. Plant Material

The aerial part (flowers, leaves, and stems) of *Cistus monspeliensis* was harvested during the month of May in the Rif region of northern Morocco. *Cistus monspeliensis* was identified by a team of botanists in the Laboratory of Biodiversity and Natural Substances at Ibn Tofail University. This collected sample was cleaned, dried in a ventilated kiln at 40°C for 24 hours, and then kept in vials away from light until use.

### 2.2. Extraction and GC/MS Analysis of Essential Oil

The extraction of *Cistus monspeliensis* essential oil was carried out by hydrodistillation using a Clevenger-type apparatus. Briefly, 250 g of dry plant material is immersed in 1 L of distilled water in a 2 L flask [17]. The mixture is brought to the boil for 4 h. The recovered essential oil is stored away from light at +4°C until analysis.

The analysis of the *Cistus monspeliensis* essential oil (EOE) extract was carried out by coupling GC/MS at the University Center for Analysis, Expertise, Technology Transfer and Incubation at Ibn Tofail University of Kenitra. The apparatus used was of Bruker brand consisting of a chromatogram of type 456-GC coupled to a mass spectrometer of type EVOQ TQ operating in electronic impact mode. The capillary column used is an Rxi-5Sil MS (30 m × 0.25 mm ID × 0.25 *μ*m df). The oven temperature is initially maintained at 60°C for 5 min, with an increase of 10°C/min to 300°C where it remains 10 minutes. The ionization energy is set at 70 eV, the temperature of the injector at 280°C, and that of the ion source at 250°C. The carrier gas flow rate (Helium) was 1 mL/min and the injection volume was 1 *μ*L.

The identification of the structures of the compounds was confirmed by comparing the mass spectra obtained with those of the pure compounds by referring to the mass spectra library of the device (NIST 2014).

### 2.3. Extraction of Phenolic Compounds

To extract the phenolic compounds, the first operation carried out was the elimination of nonpolar compounds (lipids) soluble in hexane. The dried and milled plant was extracted with a Soxhlet apparatus using hexane as the extraction solvent. The residue obtained was then macerated with a solvent mixture (water/acetone: 30/70, v/v) for 2 hours at ambient temperature. The mixture obtained was filtered, then the filtrate was concentrated with Rotavapor. This operation was repeated twice in order to exhaust the residue. The product obtained was lyophilized and the water-acetone extract (WAE) is obtained [9].

**Figure 1:**
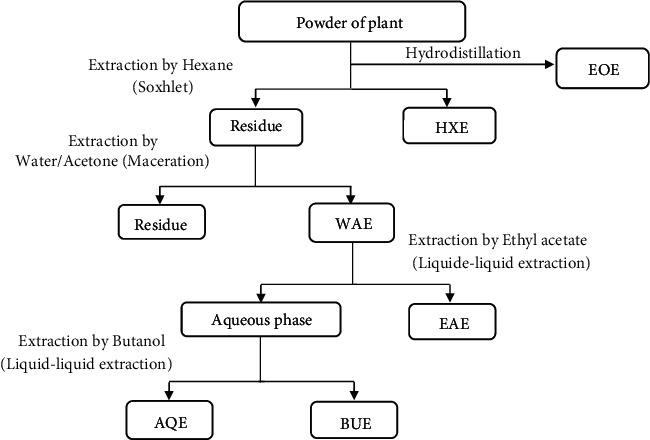
*Cistus monspeliensis* extraction protocol. EOE: essential oil; HXE: hexanoic extract; WAE: water/acetone extract; EAE: ethyl acetate extract; BUE: butanoic extract; AQE: aqueous extract.

In order to separate the phenolic compounds, the WAE extract was solubilized in distilled water and underwent liquid/liquid extractions in different solvents of increasing polarity. Three types of extracts were obtained using successively the ethyl acetate (EAE), the butanol (BUE) as extraction solvent, and the aqueous extract (AQE). The extraction protocol used is summarized in Figure 1.

### 2.4. Phytochemical Screening

The phytochemical tests consist of detecting different families of existing compounds in *Cistus monspeliensis* by qualitative reactions. The detection of these chemical compounds is based on precipitation reactions, a specific color change, or examination under ultraviolet light.

#### 2.4.1. Test of Tannins

The presence of tannins was demonstrated by adding 1 mL of each extract, 1 mL of distilled water, and 2 drops of a ferric chloride (FeCl_3_, 2%) solution; a positive test is revealed by the appearance of a blue color in the presence of hydrolysable tannins and greenish blue in the presence of condensed tannins [18]. The differentiation between the hydrolysable and condensed tannins is carried out by the Stiasny reagent (formaldehyde/concentrated hydrochloric acid 2 : 1, v/v) depending on the following operational methods [19].

*(1) Condensed Tannins.* To 1 mL of each extract solution, 1 mL of Stiasny reagent was added; the resulting mixture was heated at 90°C for 15 min. The appearance of a precipitate shows the existence of condensed tannins.

*(2) Hydrolysable Tannins.* To reveal the presence of the hydrolysable tannins, the previously heated mixture is filtered. 0.5 mL of the filtrate is saturated with sodium acetate; to this mixture three drops of a solution of (FeCl_3_, 2%) are added. The appearance of a blue-black color shows the existence of hydrolysable tannins not precipitated by the Stiasny reagent.

#### 2.4.2. Test of Flavonoids

Flavonoid detection reaction consists of putting 5 mL of the extract solution in a tube to which 1 mL of concentrated HCl and a few fragments of magnesium turnings are added. The presence of the flavonoids is highlighted by the appearance of a red, orange, or pink color [20].

#### 2.4.3. Test of Saponins

The saponins are characterized by a foam index. Test tubes containing 2 mL of the extract and 2 mL of distilled water are added; the mixture is subjected to considerable agitation. The mixture is left for 20 minutes and the saponins presence is evaluated by measuring the height of the formed foam [21].

- No foam: negative test (−)
- Foam less than 1 cm high: weakly positive test (+)
- Foam of height between 1 cm and 2 cm: positive test (++)
- Foam more than 2 cm high: very positive test (+++)

#### 2.4.4. Test of Reducing Sugars

The detection of reducing sugars happens by treating 1 mL of the extract to be analyzed with 2 mL of distilled water and 20 drops of Fehling liquor and then heating in a water bath. A positive test is indicated by the occurrence of a brick-red precipitate [22].

#### 2.4.5. Test of Glycosides

In a test tube 1 mL of acetic acid, 1 mL of concentrated sulfuric acid and then 3 drops of 2% FeCl_3_ are added to 1 mL of the extract solution. The occurrence of a blue-green color or a brown ring shows the existence of glycosides [23].

#### 2.4.6. Test of Steroids and Triterpenes

5 mL of the extract, dissolved in chloroform, is added to 1 mL of acetic anhydride; then, 0.5 mL of concentrated H_2_SO_4_ is added to the bottom of the tube without shaking. The formation of a brownish-red ring at the contact area of the two liquids confirms the presence of triterpenes, while the dark green turn of the supernatant layer (aqueous phase) reveals the presence of steroids in the extract [24].

### 2.5. Determination of Phenolic Compounds

#### 2.5.1. Quantification of Total Polyphenols

The quantitative study of the total polyphenols of the various extracts is carried out by the method using the Folin-Ciocalteu reagent [25]. In test tubes, a volume of 0.2 mL of each sample dilution of the standard (gallic acid) is added to 1 mL Folin-Ciocalteu reagent diluted 10 folds. After 2 min, 0.8 mL of Na_2_CO_3_ (7.5%) is added. A control is prepared in parallel, under the same conditions, with distilled water instead of the extract solution; then, the entire sample is incubated for half an hour at 25°C and the absorbance is read at 765 nm.

The determination of total polyphenols content was performed by means of the calibration line (*y* = *ax* + *b*) obtained by the reading of the optical density as a function of the concentrations of the gallic acid. The polyphenol content is expressed in mg equivalent of gallic acid per gram of dry matter (mg EGA/g DM).

#### 2.5.2. Quantification of Flavonoids

The assessment of the flavonoid content of the extracts is performed by the method based on the use of the aluminum trichloride reagent [26]. 1 mL of each sample dilution or quercetin (used as standard) is blended with 3 mL of distilled water. After manual agitation, 0.3 mL of sodium nitrate (5%) is added and the mixture is agitated well. After 5 min, 0.2 mL of aluminum chloride is added to the mixture; then, the whole thing is incubated in the darkness at ambient temperature for half an hour; afterward 0.5 mL of sodium hydroxide (1 M) is added. After that the optical density is determined at 510 nm.

The reading of the optical density makes it possible to determine the concentration of flavonoids in each solution by referring to a calibration curve drawn from the quercetin solutions. This concentration is expressed in mg equivalent of quercetin per gram of dry matter (mg EQ/g DM).

#### 2.5.3. Quantification of Hydrolysable Tannins

The hydrolysable tannin assay is carried out by the method described by Çam et al. [27]; this method is based on the reaction of potassium iodate with the galloyl esters, which produces a red intermediate whose concentration can be measured by a spectrophotometer at 550 nm. To 1 mL of each dilution of the extracts and to the tannic acid (used as standard), a volume of 5 mL of 2.5% KIO_3_ is added and a blank is prepared in parallel. After 5 min of incubation, the absorbance of the red color mixture is determined at a wavelength of 550 nm.

The concentration of hydrolysable tannins was determined thanks to the regression equation of the calibration curve (*y* = *ax* + *b*). This concentration is expressed in milligram of tannic acid equivalent per gram of the dry matter (mg ETA/g DM).

#### 2.5.4. Quantification of Condensed Tannins

This test is based on the condensation of condensed tannins with vanillin in an acid medium [9]. The vanillin test is done by mixing 0.5 mL of the solution of each extract with 3 mL of the vanillin-MeOH solution [4% (m/v)] and 1.5 mL of concentrated HCl; then the mixture is incubated at darkness at ambient temperature for 15 min. The absorbance of each solution is measured at 500 nm using a spectrophotometer; three optical density measurements are determined for each solution.

The content of condensed tannins, expressed in mg equivalent of catechin per gram of dry matter (mg EC/g DM), was determined using the calibration curve (*y* = *ax* + *b*) obtained from the catechin solutions at different concentrations.

### 2.6. Evaluation of the Antioxidant Power of Extracts of *Cistus monspeliensis*

The antioxidant power is not based on only one model of antioxidant test. Practically, many methods are used, and most of them are based on the coloration or discoloration of a given reagent in the reaction medium. In our study, we choose to apply the free radical reduction method DPPH (2,2-DiPhenyl-1-Picryl-Hydrazyl) and the iron reduction test (or FRAP: Ferric Reducing Antioxidant Power).

#### 2.6.1. DPPH Free Radical Reducing Test

The protocol utilized to assess the antioxidant power of the different extracts is that described by Sanchez-Moreno [28]. It consists of preparing solutions of increasing concentrations by dilution of the mother solution of the different extracts and ascorbic acid taken as standard. The DPPH solution is prepared by dissolving 6 mg of DPPH in 200 mL of ethanol. In test tubes, 2 mL of the freshly prepared DPPH solution is added to 0.1 mL of each solution. A blank is prepared in parallel with methanol. Then the whole thing is incubated in the darkness for 30 minutes. The absorbance is measured at 517 nm, and three optical density measurements are determined for each solution.

The assessment of the antioxidant power is expressed as a percentage of inhibition of the DPPH radical depending to the following formula [29]:

(1) $$\begin{array}{c} \%\text{ of inhibition}=\frac{\text{Ab}-\text{As}}{\text{Ab}}\;\times 100, \end{array}$$

where Ab is the absorbance of the blank and As is the absorbance of the sample.

This formula allowed to trace the straight line (*y* = *ax* + *b*) representing the variation of the percentage of inhibition of each sample. From this straight line, it is possible to determinate the concentration that reduces 50% of DPPH in each sample studied and ascorbic acid. This concentration, called IC50, is usually determined by the following equation:

(2) $$\begin{array}{c} \text{IC}50=\frac{50-b}{a}. \end{array}$$

*a* is slope of the line; *b* is intercept of the line.

#### 2.6.2. Ferric Reducing Antioxidant Power (FRAP)

The reducing capacity of the ferric ion (Fe^3+^) of the extracts is evaluated according to the protocol described by Oyaizu [30]. Stock solutions of known concentrations are prepared by dissolving the various extracts (WAE, BUE, and EAE) in methanol and the aqueous extract (EAQ) in distilled water, then making appropriate dilutions to prepare solutions in a range of increasing concentrations. The solutions of ascorbic acid (used as standard) are prepared in the same way. In test tubes each containing 1 mL of sample solution, 2.5 mL of phosphate buffer (0.2 M, pH 6.6) is added followed by 2.5 mL of potassium hexacyanoferrate [(K_3_Fe(CN)_6_), 10 g/L]. This mixture is heated at 50°C in a water bath for 20 minutes. 2.5 mL of trichloroacetic acid is then added and the mixture is centrifuged at 3000 rpm for 10 minutes. Finally, to 2.5 mL of supernatant, 2.5 mL of distilled water then 0.5 mL of ferric chloride [(FeCl_3_), 1 g/L] are added. A blank is prepared under the same operational conditions. The reading of the optical density is performed at 700 nm, and three measurements are determined for each solution.

The antioxidant power of the extracts is calculated using the standard curves obtained with ascorbic as standard. The results are obtained by determination of IC_0.5_ which shows that the concentration is equivalent to the absorbance 0.5 [31].

## 3. Results and Discussion

### 3.1. Quantitative Results of *Cistus monspeliensis* Extracts

Five extracts of different appearance were obtained: the hexanic (HXE), water-acetone (WAE), ethyl acetate (EAE), butanoic (BUE), and aqueous (AQE) extracts.

From 30 g of dry and finely ground *Cistus monspeliensis*, we obtained approximately 8.6% of HXE extract and 27.6% of WAE extract. Three times more polar products are obtained than nonpolar (or lipids) products. The separation by liquid-liquid extraction of the WAE extract provided 59.6% of AQE extract, which is approximately three times the amount of the EAE extract (19.2%) and that of the BUE extract (20.2%) (Table 1).

Extraction by hydrodistillation of 250 g of *Cistus monspeliensis* dry matter provided 350 mg of the extract (EOE), which represents a yield equal to 0.14% of essential oil. This value is comparable with previous results obtained on the same species of *Cistus* [32].

### 3.2. Results of GC/MS Analysis

**Figure 2:**
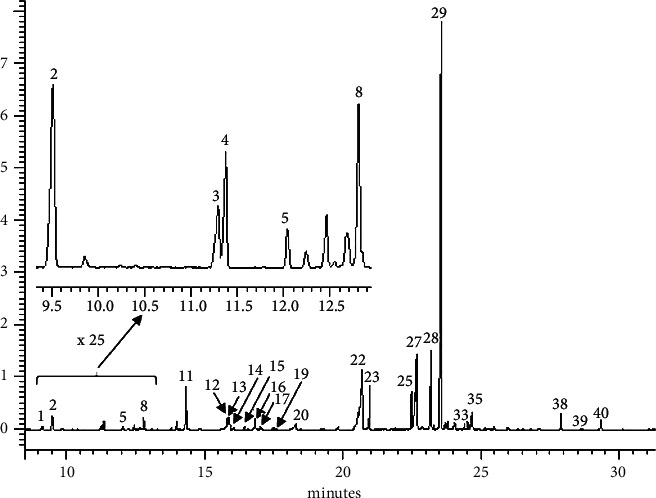
Chromatogram obtained using TIC (total ion counts).

Thanks to the analysis by GC/MS coupling and following the in-depth study of the mass spectra obtained, we were able to identify twenty compounds, which represent around 92% of *Cistus monspeliensis*. The chromatogram obtained is shown in Figure 2 and the chemical structures of the identified compounds as well as their percentages are represented in Table 2.

The major compound identified is the oxide of 13 epi-manoyl oxides; it is a diterpene of the labdane type. Its presence in significant proportions (49%) in essential oil is an interesting result from a pharmacological point of view because the antimicrobial activity of certain essential oils has been attributed to the presence of manoyl derivatives. Generally, labdanes are used in perfumery as fixatives. They have also been used for their therapeutic effects against hair loss, coughs, catarrh, and asthma, as well as for the healing of cancer and ulcers [33]. These compounds can be classified into different families:

- Diterpenes (74.72 mg/100gDM): 13-epi-manoyl oxide and 3,3a, 6,6,9a-Pentamethyldodecahydro-3,9b-epoxycyclopenta [a] naphthalene.
- Norisoprenoids (4.53 mg/100gDM): dihydro-*β*-ionone; *β*-ionone; and vitispirane.
- Aromatic compounds (7.64 mg/100gDM): benzaldehyde; phenylacetaldehyde; 4-ethyl-1,2-dimethylbenzene; 1-phenyl-1,3-butadiene; 4-hydroxy-3-methyl acetophenone; 4- (4-methylphenyl) pentanal; and 1- (6,10-dimethylundeca-5,9-dien-2-yl) -4-methylbenzene.
- Other nonaromatic oxygenates (6.64 mg/100gDM): myrtenyl acetate; 2,4,4-trimethylcyclopentanone; nonanal; 6-methyl-3,5-heptadien-2-one; 6,10,14-trimethylpentadecan-2-one; and 4-caranol.
- Saturated fatty acids (30.27 mg/100gDM): decanoic acid; lauric acid; myristic acid; and palmitic acid.
- Hydrocarbons (4.64 mg/100gDM): pentacosane; hexacosane; heptacosane; and octacosane.
- Some previous studies on the chemical composition of the essential oil of *Cistus monspeliensis* presented qualitative and quantitative results clearly different from those which we obtained. As an example, we may mention the study made by Viuda-Martos et al. [34], in which they identified as main components 1,8-cineole (8.8%), bornyl acetate (8, 7%), and *α*-pinene (5.6%); these compounds are completely absent in our case, while 13-epi-manoyl oxide, clearly predominant in our essential oil, has not been detected. In another study carried out on the same plant originating from the Island of Grete in Greece [35], the authors detected as main compounds 13-epi-manoyl oxide (39.69%) and kaur-16 -ene (18.51%); the latter was not detected in our sample. However, the results obtained by Oller-López et al. [32] indicate that 13-epi-manoyl oxide (7.7%), 6,10,14-trimethylpentadecan-2-one (4.3%), and epi-manool-13 (3.9%) were the main components. This is qualitatively consistent with our results. In addition, other studies have also shown the presence, in this essential oil, of some fatty acids and hydrocarbons [34–36]. The authors affirmed that the qualitative and quantitative differences observed, in the chemical composition of *Cistus monspeliensis* essential oil, could be attributed to the variability of the climatic and soil conditions, at the stage of the vegetative cycle, and to seasonal variations.

### 3.3. Results of Phytochemical Tests

The phytochemical tests make it possible to highlight the existence of some chemical compounds by simple qualitative reactions. The phytochemical characterization tests performed on the various extracts gave the results exposed in Table 3.

From these results, it is noted that all the extracts contain hydrolysable tannins and glycosides; the latter are probably bound as hydrolysable tannins, or to other compounds such as saponins. The WAE extract responded positively to all the tests, and its separation provided different results by their polyphenols composition.

Previous phytochemical studies on *Cistus monspeliensis* have revealed the presence of several compounds, such as flavonoids, hydrolysable tannins, condensed tannins, and saponins [37–40], which confirms our results.

Lipid extracts generally consist of hydrocarbons, fatty acids, and other aliphatic and oxygenated compounds. The results of phytochemical tests carried out on the lipid extract (HXE) show that it contains steroids and triterpenes.

### 3.4. Results of the Quantification of Phenolic Compounds

#### 3.4.1. Total Polyphenol Content

The content of total polyphenols of the different extracts of *Cistus monspeliensis* is assessed by the Folin-Ciocalteu method. Gallic acid was utilized as a standard and allowed us to trace the calibration line. The results obtained are expressed in milligrams of equivalents of gallic acid per gram of dry matter (mg EGA/g DM), using the linear regression equation of the calibration line (available here) (Appendix).

These results, illustrated in Table 4, show that a total of 131.42 mg EGA/g DM is obtained of phenolic compounds, which represents a very interesting value. The extracts obtained by separation of the WAE extract contain very appreciable polyphenol contents, particularly the AQE extract with a content of 97.15 mg EGA/g DM.

According to Tawaha, et al. [41], who reported that a total phenolic compounds content more than 20 mg EGA/g dry weight could be considered very high, it can be considered that all the extracts of *Cistus monspeliensis* studied have a very good origin of phenolic compounds. In a precedent study, a total polyphenol content of 79.19 ± 2.42 mg EGA/g DM was obtained in a polyphenols assay of the ethanolic extract of *Cistus monspeliensis* from the city of Ouazzane located in northern Morocco [42].

#### 3.4.2. Flavonoids Content

The flavonoid content of the extracts is estimated by the method using aluminum trichloride (AlCl_3_). The quercetin, a well-known flavonoid from the flavanol family, is used as a standard and allowed us to trace the calibration line. Table 4 shows the flavonoid contents expressed in mg equivalent of quercetin per gram of dry matter (mg EQ/g DM) calculated using the linear regression equation of the calibration line (Appendix).

The flavonoid content of the extracts is significantly different; the WAE extract is rich of flavonoids with a value of 69.81 ± 0.22 mg EQ/g DM. After this separation, a high content (82.50 ± 0.89 mg EQ/g DM) is obtained in the AQE aqueous extract even if the phytochemical test on this extract has a negative response; this result is probably due to the capacity of the trichloride of aluminum compared to other complex nonflavonoid compounds. The lowest grade of 15.68 ± 0.11 mg EQ/g DM is recorded in the EAE extract.

In the same study cited before [42], the ethanolic extract of *Cistus monspeliensis* harvested from the city of Ouazzane provided a flavonoid content of the order of 19.43 mg EQ/g DM.

#### 3.4.3. Hydrolysable Tannin Content

The content of hydrolysable tannins is estimated by the method described by Çam and Hışıl [27]: the tannic acid used as a positive control made it possible to draw the calibration line (Appendix). The absorbance was read at a wavelength of 550 nm.

Thanks to the calibration line, the hydrolysable tannin content is calculated for each extract (Table 4). The results obtained are expressed in milligram of tannic acid equivalent per gram of dry matter (mg ETA/g DM).

The hydrolysable tannin contents in the extracts are significantly different, and the WAE extract contains a fairly high content of the order of 61.86 ± 0.89 mg ETA/g DM. After separation, the highest content of these compounds is obtained in the aqueous extract, presumably because of their high solubility in water.

These results attract attention to the richness of our extracts of phenolic compounds responsible for several pharmacological activities and may explain why this plant is considered as medicinal plant [13].

#### 3.4.4. Condensed Tannin Content

Quantification of condensed tannins is achieved thanks to the method using vanillin [9]. Catechin taken as standard allowed us to draw the calibration line (Appendix). Using the regression equation (*y* = 1.581*x* + 0.024) of the calibration straight which is drawn from a series of increasing concentration solutions of catechin, we calculated the content of condensed tannins in each extract (Table 4). This value is expressed in milligrams equivalents of catechin per gram of dry matter (mg EC/g DM).

The WAE extract is rich in condensed tannins; this richness is translated by an interesting content (70.05 ± 1.61 mg EC/g DM). After separation, their content varies proportionally as a function of the polarity of the extraction solvent; hence, the highest content is obtained in the aqueous extract.

### 3.5. Results of the Evaluation of Antioxidant Activity

The antioxidant power of the plant extracts is evaluated by two methods: the DPPH (2,2-diphenyl-1-picrylhydrazyle) free radical scavenging test and the FRAP (Ferric Reducing Antioxidant Power) iron reduction method.

#### 3.5.1. DPPH Free Radical Reducing Test

The antioxidant activity is defined as a proportion of DPPH free radical scavenging. Since there is no absolute measure of the antioxidant power of a compound, results are compared to ascorbic acid as the reference antioxidant. The results obtained allowed us to draw curves with an exponential rate representing the variation of the absorbance depending on concentration of the samples. The same curves are obtained for the extracts of *Cistus monspeliensis*. From these results, we were able to determine the percent of inhibition of DPPH for each solution and plot their variation as a function of the concentrations.

**Figure 3:**
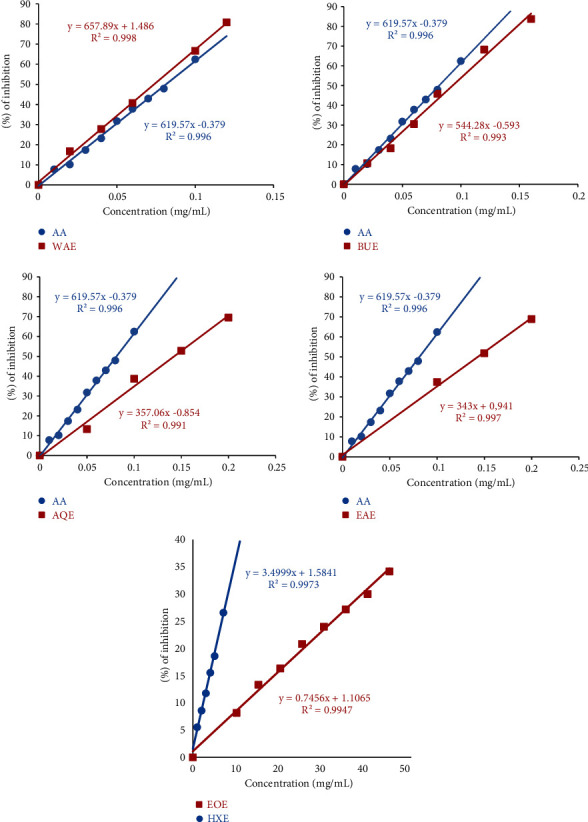
Comparison of the percentage change in DPPH inhibition as a function of the concentrations of each extract of *Cistus monspeliensis* relative to ascorbic acid. WAE: water/acetone extract; EAE: ethyl acetate extract; BUE: butanolic extract; AQE: aqueous extract; EOE: essential oil; HXE: hexanic extract; and AA: ascorbic acid.

In the same way, we were able to determine the percentages of inhibition of DPPH as a function of the different concentrations of the extracts (EO, HE, WAE, EAE, AQE, and BUE) (Figure 3), which allowed us to know the necessary concentrations to reduce 50% of the DPPH radical (IC50) of each extract. IC50 is conversely proportional to the antioxidant ability of a compound. The lowest IC50 inhibitory concentration value shows the strongest antioxidant capacity of a compound. The values of the inhibitory concentrations of ascorbic acid and each extract of *Cistus monspeliensis* are illustrated on Table 5.

From the results shown in Table 5, it is found that the ascorbic acid has a powerful antioxidant activity translated by a low inhibitory concentration of the order of 0.08 ± 0.018 mg/mL.

The results obtained for extracts of *Cistus monspeliensis* show that they are active and possess interesting antioxidant powers. WAE extract is the most active extract with a low IC_50_ value of the order of 0.079 ± 0.009 mg/mL. This very interesting result, which exceeds the antioxidant power of ascorbic acid, is probably due to its richness in various phenolic compounds confirmed by phytochemical tests. The BUE extract, which is derived from the separation of the WAE extract, also has a significant antioxidant activity with an IC_50_ value of 0.097 ± 0.006 mg/mL. This extract, too, responded positively to all phytochemical tests for phenolic compounds.

According to the results recorded, it is deduced that *Cistus monspeliensis* extracts have a high antioxidant power; this is thanks to the presence of the antioxidant molecules as flavonoids and tannins which reduce the radical DPPH because of their capacity to release hydrogen atoms [43].

In a previous study, the methanolic extract of *Cistus monspeliensis* originating in Tunisia recorded an antioxidant power (IC_50_ = 3 ± 0.36 mg/mL) against (IC_50_ = 12 ± 0.13 mg/mL) that of BHT taken as reference [14].

In addition to these results, the extract (HXE) recorded a low antioxidant activity for the two tests (DPPH and FRAP) respectively (IC_50_ = 15.03 ± 2.19 mg/mL and IC_0.5_ = 2.71 ± 0.05 mg/mL). *Cistus monspeliensis* essential oil (EOE) has the lowest activity (IC_50_ = 70.53 ± 7.01 mg/mL); this may be due to its chemical composition mainly consisting of 13-epi-manoyl oxide (49%). This result is in agreement with a previous study on the same plant native to Tunisia, in which the essential oil represents an IC_50_ value of the order of 991.9 ± 4.4 *μ*g/mL against 5.0 ± 0.8 *μ*g/mL of ascorbic acid [44].

#### 3.5.2. Ferric Reducing Antioxidant Power (FRAP)

The evaluation of the antioxidant power of extracts of *Cistus monspeliensis* by the reduction of iron (FRAP) method is based on the reduction of Fe^3+^ ferric ions to Fe^2+^ ferrous ions. The existence of ferrous ions can be assessed by measuring and observing the increase in the density of the blue color in the reaction medium at 700 nm.

**Figure 4:**
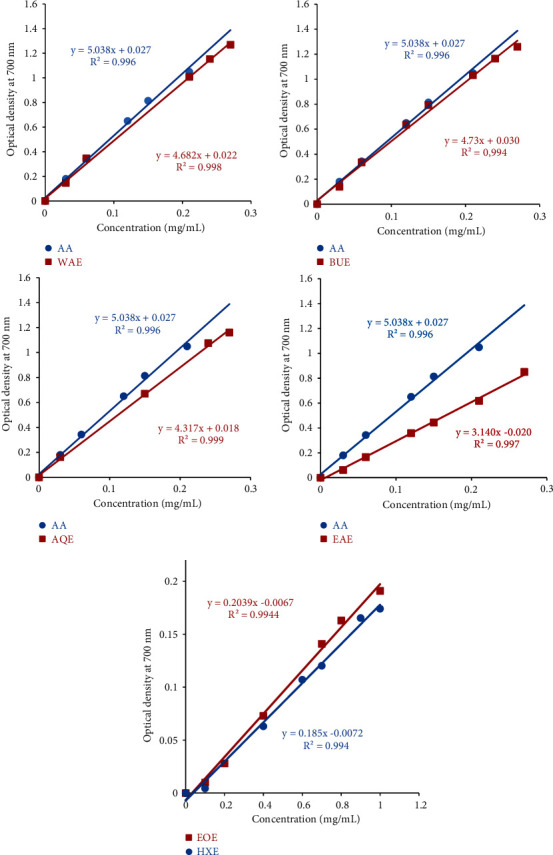
Comparison of variation of the optical density as a function of the concentrations of each extract relative to that of ascorbic acid for FRAP test. WAE: water/acetone extract; EAE: ethyl acetate extract; BUE: butanolic extract; AQE: aqueous extract; EOE: essential oil; HXE: hexanic extract and AA: ascorbic acid.

The evaluation of the antioxidant power of extracts of *Cistus monspeliensis* is compared with that of the ascorbic acid, obtained by plotting the optical density read as a function of concentration of ascorbic acid.  Figure 4 shows the comparison of the straight line obtained for each extract with that of ascorbic acid.

Table 5 shows the values of the IC_50_ concentrations obtained for ascorbic acid and for each extract of *Cistus monspeliensis*. From these results, we can see that all extracts studied are active. In addition, BUE and WAE extracts showed the greatest ability to reduce iron with IC_50_ values of 0.099 ± 0.089 mg/mL and 0.102 ± 0.059 mg/mL, respectively. We thus find the same results recorded previously in the DPPH reduction study.

In contrast, as in the case of the DPPH test, the essential oil (EOE) and the hexanic extract (HXE) recorded the lowest ferric reducing power, with IC_0.5_ values of 2.50 ± 0.06 and 2.71 ± 0.05 mg/mL, respectively.

## 4. Conclusions

The present study allowed us to highlight the presence of bioactive compounds in *Cistus monspeliensis* extracts: on the one hand, the essential oil consists mainly of 13-epi-manoyl oxide, which is an interesting compound from a pharmacological point of view. On the other hand, polar extracts are rich in polyphenols, flavonoids, tannins, saponins, etc., endowed with antioxidant properties.

The estimation of total polyphenol, flavonoid, hydrolysable tannin, and condensed tannin contents was determined by known calorimetric assay methods.

The antioxidant activity of *Cistus monspeliensis* extracts was evaluated by two different methods: the DPPH radical reduction test and the FRAP iron reduction test. The results obtained show that the extracts studied have a real and potential interest thanks to their powerful antioxidant power. To know the constituents responsible for this antioxidant activity, other analytical techniques (such as HPLC-MS) must also be implemented in future research. *Cistus monspeliensis* native to northern Morocco can be a source of natural antioxidants used in the food industry and replace chemical antioxidants.

More advanced studies in vivo on antidiabetic, anti-inflammatory, and antiproliferative activity would be necessary to better understand the mechanism of action of the bioactive molecules of this plant, their therapeutic dose, and their site of action at the cell level. This would make it possible to prepare pharmaceutical products of great therapeutic interest.

## Figures and Tables

**Table 1:** Quantitative results of extractions.

Extract	Extraction of the plant			Separation of WAE		
	HXE	WAE	Residue	EAE	BUE	AQE
Mass obtained in (g)	2.59	8.28	17.21	1.65	1.68	4.94
Yield (%)	8.64^*∗*^	27.63^*∗*^	57.36^*∗*^	19.2^*∗∗*^	20,2^*∗∗*^	59.6^*∗∗*^

^*∗*^Yield expressed as % of the mass of the extract relative to that of the dry matter. ^*∗∗*^Yield expressed as % of the mass of the extract relative to that of the WAE extract.

**Table 2:** Chemical composition of the essential oil from *Cistus monspeliensis* analyzed by GC/MS.

N°	Compound	Chemical structure	RT (min)	RI	Amount (mg/100gDM)
1	Benzaldehyde	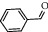	9.13	1296	0.45
2	2,4,4-Trimethylcyclopentanone	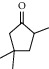	9.51	1322	1.93
3	Nonanal	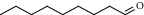	11.29	1446	0.62
4	Phenylacetaldehyde	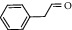	11.37	1451	0.90
5	6-Methyl-3,5-heptadien-2-one	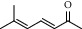	12.04	1498	0.29
6	m/z 153, 109, 95, 81, 67, 55, 43	NI	12.46	1527	0.39
7	m/z 151, 110, 91, 83, 67, 55, 43	NI	12.68	1542	0.41
8	1-Phenyl-1,3-butadiene	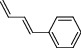	12.80	1551	1.20
9	m/z 162, 126, 107, 91, 67, 55, 41	NI	13.81	1620	0.21
10	m/z 164, 151, 126, 111, 95, 79, 70, 55, 41	NI	13.99	1633	0.85
11	Vitispirane	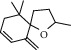	14.34	1658	3.47
12	4-ethyl-1,2-dimethylbenzene	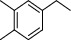	15.84	1762	1.72
13	Decanoic acid	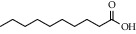	15.89	1765	1.55
14	4-Hydroxy-3-methyl acetophenone	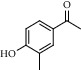	16.07	1778	0.17
15	Myrtenyl acetate	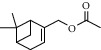	16.44	1803	0.24
16	Dihydro-*β*-ionone	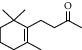	16.84	1831	0.88
17	4-(4-Methylphenyl) pentanal	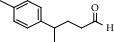	17.02	1844	0.25
18	m/z 216, 180, 85, 71, 57, 43	NI	17.50	1877	0.18.
19	*β*-Ionone	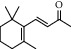	17.61	1885	0.18
20	Lauric acid	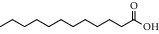	18.32	1934	0.91
21	m/z 205, 119, 117, 91, 77, 68, 55, 43	NI	19.85	2020	0.18
22	Myristic acid	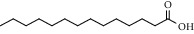	20.71	2100	17
23	6,10,14-Trimethylpentadecan-2-one	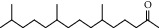	20.97	2118	3.14
24	m/z 272, 228, 190, 121, 91, 87, 74, 55, 43	NI	21.89	2182	0.18
25	1-(6,10-Dimethylundeca-5,9-dien-2-yl)-4-methylbenzene	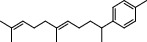	22.49	2224	2.95
26	m/z 284, 256, 213, 119, 91, 73, 55, 43	NI	22.61	2232	4.65
27	Palmitic acid	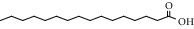	22.68	2237	10.81
28	3,3a,6,6,9a-Pentamethyldodecahydro-3,9b-epoxycyclopenta[a]naphthalene	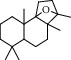	23.19	2272	6.72
29	13-Epi-manoyl oxide	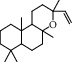	23.58	2299	68
30	m/z 228, 199, 146, 132, 119, 91, 67, 55, 43	NI	23.72	2309	0.84
31	m/z 254, 240, 189, 119, 105, 91, 79, 67, 55, 41	NI	24.02	2329	0.42
32	m/z 272, 209, 189, 105, 91, 79, 67, 55, 41	NI	24.06	2332	0.53
33	4-Caranol	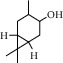	24.41	2357	0.42
34	m/z 242, 196, 135, 123, 107, 95, 81, 69, 55, 43	NI	24.52	2364	0.66
35	Pentacosane	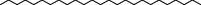	24.70	2377	2.83
36	m/z 292, 191, 124, 85, 71, 58, 43	NI	25.49	2431	0.28
37	m/z 284, 150, 119, 91, 67, 55, 43	NI	25.97	2465	0.17
38	Hexacosane	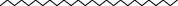	27.91	2600	1.04
39	Heptacosane	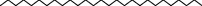	28.62	2649	0.07
40	Octacosane	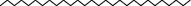	29.35	2700	0.70

NI: not identified; RT: retention time; RI: retention index.

**Table 3:** Results of phytochemical tests carried out on the different extracts.

Phytoconstituents	HXE	WAE	EAE	BUE	AQE
Steroids and triterpenes	+	−	−	−	−
Hydrolysable tannins	−	+	+	+	+
Condensed tannins	−	+	−	+	+
Flavonoids	−	+	+	+	−
Saponins	−	+	−	−	+++
Reducing sugars	−	+	−	+	+
Glycosides	−	+	+	+	+

(+), presence; (−), absence. WAE, water-acetone extract; EAE, ethyl acetate extract; BUE, butanoic extract; AQE, aqueous extract.

**Table 4:** The content of total polyphenols (CTP), of flavonoids (CF), of hydrolysable tannins (CHT), and of condensed tannins (CCT), recorded in the different extracts.

Extract	CTP (mg EGA/g DM)	CF (mg EQ/g DM)	CHT (mg ETA/g DM)	CCT (mg EC/g DM)
WAE	131.42 ± 0.93	69.81 ± 0.22	61.86 ± 0.89	70.05 ± 1.61
EAE	20.55 ± 0.39	15.68 ± 0.11	13.33 ± 0.24	4.35 ± 0.23
BUE	31.68 ± 0.06	18.57 ± 0.17	11.25 ± 0.26	10.45 ± 0.23
AQE	97.15 ± 0.11	82.50 ± 0.89	107.45 ± 1.06	24.38 ± 0.49

WAE, water-acetone extract; EAE, ethyl acetate extract; BUE, butanoic extract; AQE, aqueous extract.

**Table 5:** Results of the evaluation of the antioxidant activity of the different extracts.

Extract	DPPH ^*∗*^IC_50_ (mg/mL)	FRAP ^*∗∗*^IC_0.5_ (mg/mL)
AA	0.08 ± 0.018	0.096 ± 0.003
WAE	0.079 ± 0.009	0.097 ± 0.005
EAE	0.164 ± 0.030	0.167 ± 0.010
BUE	0.097 ± 0.006	0.099 ± 0.012
AQE	0.159 ± 0.024	0.101 ± 0.015
HXE	15.03 ± 2.19	2.71 ± 0.05
EOE	70.53 ± 7.01	2.50 ± 0.06

^*∗*^Concentration of the extract, which can reduce 50% of the DPPH radical. ^*∗∗*^Concentration equivalent to the absorbance 0.5.

## Data Availability

All the data are included within the manuscript.

## Supplementary Materials

Supplementary Materials Appendix: The analysis of the *Cistus monspeliensis* essential oil (EOE) extract was carried out by coupling GC/MS at the University Center for Analysis, Expertise, Technology Transfer and Incubation at the Ibn Tofail University of Kenitra. The apparatus used was of Bruker brand consisting of a chromatogram of type 456-GC coupled to a mass spectrometer of type EVOQ TQ operating in electronic impact mode. The capillary column used is an Rxi-5Sil MS (30 m × 0.25 mm ID × 0.25 *μ*m df). The oven temperature is initially maintained at 60°C for 5 min, with an increase of 10°C/min to 300°C where it remains 10 minutes. The ionization energy is set at 70 eV, the temperature of the injector at 280°C, and that of the ion source at 250°C. The carrier gas flow rate (Helium) was 1 mL/min and the injection volume was 1 *μ*L. The identification of the structures of the compounds was confirmed by comparing the mass spectra obtained with those of the pure compounds by referring to the mass spectra library of the device (NIST 2014).
