# Supplementary material for: Chemical Composition of Essential Oil, Phenolic Compounds Content, and Antioxidant Activity of Cistus monspeliensis from Northern Morocco
Source: Biochem Res Int. 2021 Dec 7;2021:6669877. doi: 10.1155/2021/6669877 (PMC8670979; doi:10.1155/2021/6669877)
Supplement: Supplementary Materials — Appendix: The analysis of the Cistus monspeliensis essential oil (EOE) extract was carried out by coupling GC/MS at the University Center for Analysis, Expertise, Technology Transfer and Incubation at the Ibn Tofail University of Kenitra. The apparatus used was of Bruker brand consisting of a chromatogram of type 456-GC coupled to a mass spectrometer of type EVOQ TQ operating in electronic impact mode. The capillary column used is an Rxi-5Sil MS (30 m × 0.25 mm ID × 0.25 μm df). The oven temperature is initially maintained at 60°C for 5 min, with an increase of 10°C/min to 300°C where it remains 10 minutes. The ionization energy is set at 70 eV, the temperature of the injector at 280°C, and that of the ion source at 250°C. The carrier gas flow rate (Helium) was 1 mL/min and the injection volume was 1 μL. The identification of the structures of the compounds was confirmed by comparing the mass spectra obtained with those of the pure compounds by referring to the mass spectra library of the device (NIST 2014). [file 6669877.f1.docx]

**Detail of the chromatography**

The analysis of the *Cistus monspeliensis* essential oil (EOE) extract was carried out by coupling GC/MS at the University Center for Analysis, Expertise, Technology Transfer and Incubation at the Ibn Tofail University of Kenitra. The apparatus used was of Bruker brand consisting of a chromatogram of type 456-GC coupled to a mass spectrometer of type EVOQ TQ operating in electronic impact mode. The capillary column used is an Rxi-5Sil MS (30 m × 0.25 mm ID × 0.25 μm df). The oven temperature is initially maintained at 60 ° C for 5 min, with an increase of 10 °C/min to 300 °C where it remains 10 minutes. The ionization energy is set at 70 eV, the temperature of the injector at 280 ° C and that of the ion source at 250 °C. The carrier gas flow rate (Helium) was 1 mL / min and the injection volume was 1 μL.

The identification of the structures of the compounds was confirmed by comparing the mass spectra obtained with those of the pure compounds by referring to the mass spectra library of the device (NIST 2014).

Total ionic current (TIC) of *Cistus monspeliensis* essential oil.

**
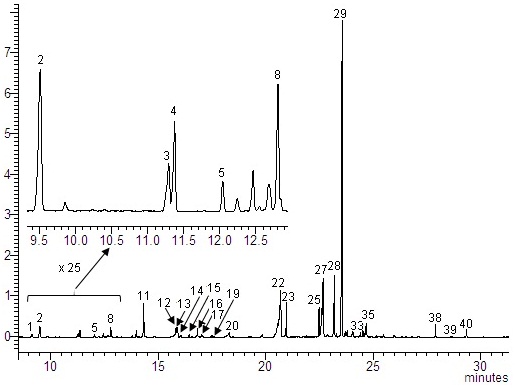
**

**Some mass spectra of essential oil compounds**

1) Benzaldehyde


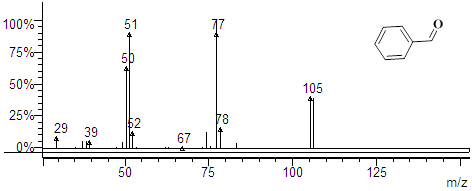


2) 2,4,4-Trimethylcyclopentanone


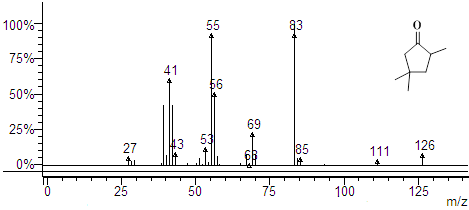


4) Phenylacetaldehyde


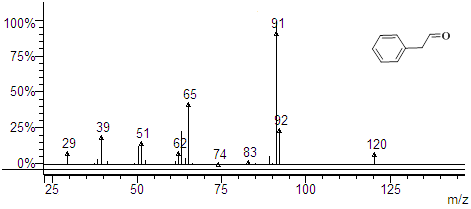


5) 6-methyl-3,5-heptadien-2-one


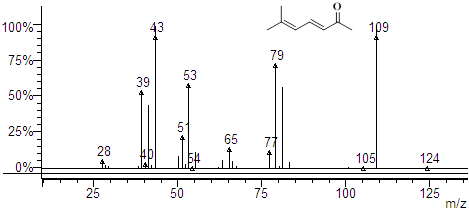


8) 1-Phenyl-1,3-butadiene


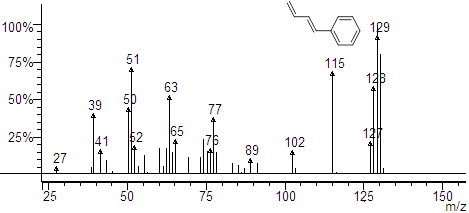


11) Vitispirane


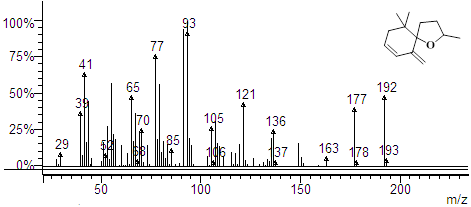


14) 4-hydroxy-3-methyl acetophenone


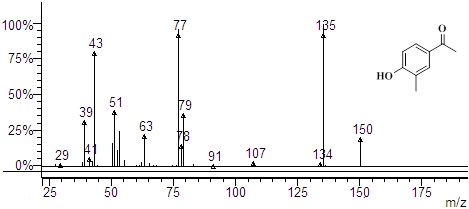


17) 4-(4-Methylphenyl) pentanal


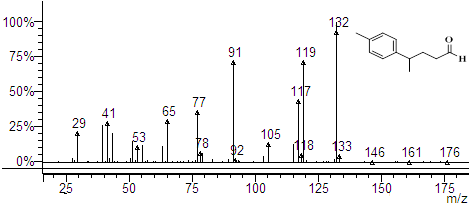


19) β-ionone


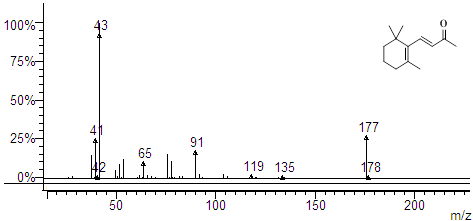


25) 1-(6,10-dimethylundeca-5,9-dien-2-yl)-4-methylbenzene


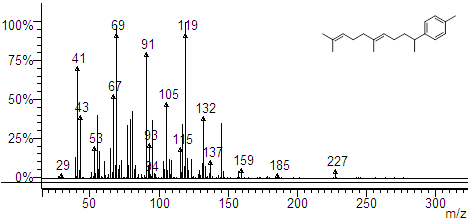


27) Palmitic acid


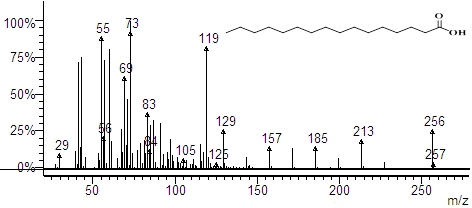


28) 3,3a,6,6,9a-Pentamethyldodecahydro-3,9b-epoxycyclopenta[a]naphthalene


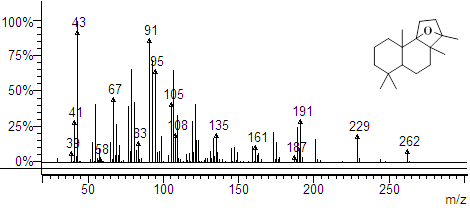


29) 13-epi-manoyl oxide


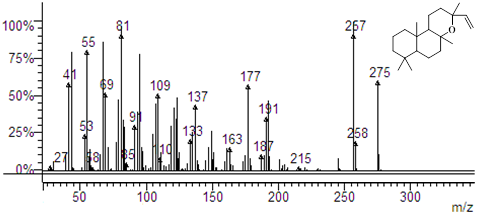


33) 4-Caranol


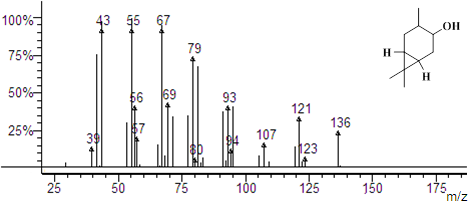


**Calibration line of standards**

1) Gallic acid

2) Quercetin

3) Tannic acid

4) Catechin
